# A Theoretical Framework to Derive Simple, Firing-Rate-Dependent Mathematical Models of Synaptic Plasticity

**DOI:** 10.3389/fncom.2019.00026

**Published:** 2019-05-08

**Authors:** Janne Lappalainen, Juliane Herpich, Christian Tetzlaff

**Affiliations:** ^1^Department of Computational Neuroscience, Third Institute of Physics-Biophysics, Georg-August-University, Göttingen, Germany; ^2^Bernstein Center for Computational Neuroscience, Georg-August-University, Göttingen, Germany

**Keywords:** STDP, synaptic plasticity, calcium, regression analysis, learning, activity-dependency

## Abstract

Synaptic plasticity serves as an essential mechanism underlying cognitive processes as learning and memory. For a better understanding detailed theoretical models combine experimental underpinnings of synaptic plasticity and match experimental results. However, these models are mathematically complex impeding the comprehensive investigation of their link to cognitive processes generally executed on the neuronal network level. Here, we derive a mathematical framework enabling the simplification of such detailed models of synaptic plasticity facilitating further mathematical analyses. By this framework we obtain a compact, firing-rate-dependent mathematical formulation, which includes the essential dynamics of the detailed model and, thus, of experimentally verified properties of synaptic plasticity. Amongst others, by testing our framework by abstracting the dynamics of two well-established calcium-dependent synaptic plasticity models, we derived that the synaptic changes depend on the square of the presynaptic firing rate, which is in contrast to previous assumptions. Thus, the here-presented framework enables the derivation of biologically plausible but simple mathematical models of synaptic plasticity allowing to analyze the underlying dependencies of synaptic dynamics from neuronal properties such as the firing rate and to investigate their implications in complex neuronal networks.

## 1. Introduction

Synaptic plasticity serves as an essential mechanism of neuronal networks being linked to diverse functional properties such as the cognitive mechanisms of learning and memory (Hebb, 1949; Martin et al., 2000). Amongst others, to understand the details of this link between synaptic plasticity and functional properties of a neuronal system, theoretical or mathematical models of synaptic plasticity are formulated and investigated (Dayan and Abbott, 2001; Gerstner et al., 2012, 2014). On the one hand, detailed biological models of synaptic plasticity are formulated closely related to experiments, which provide molecular details or synaptic dynamics given diverse stimulation protocols (Earnshaw and Bressloff, 2006; Graupner and Brunel, 2010; Graupner and Brunel, 2012; Antunes et al., 2016; Gallimore et al., 2018). However, to capture the richness of the underlying molecular processes and to match a wide repertoire of experimental findings, the resulting theoretical models become mathematically rather complex and often depend on detailed spike-timings, which impedes further analysis especially on the neuronal network level. On the other hand, to investigate the link between synaptic plasticity and functional properties of neuronal systems, compact, simple theoretical models of synaptic plasticity are developed neglecting the underlying biological details (Tsodyks and Feigelman, 1988; Gerstner and Kistler, 2002; Tetzlaff et al., 2011, 2013; Knoblauch and Sommer, 2016). In contrast to the spike-dependency of the detailed models, these compact models usually depend on the average firing rates of neurons (Gerstner and Kistler, 2002). However, the detailed formulation of these compact models are rather phenomenological and they are only loosely linked to the detailed biological models.

Here, we will provide a mathematical method enabling us to derive a compact, firing-rate-dependent theoretical model from a biologically detailed spike-based model. For the detailed model, we consider two versions of a well-established calcium-based spike-timing-dependent plasticity model (Graupner and Brunel, 2012; Graupner et al., 2016; Li et al., 2016). We will show that this model can be simplified such that the general dynamics induced by it can be well-described by a compact, rate-dependent model.

Many experimental studies have been performed to investigate the multiplicity of molecular processes, which are involved in synaptic plasticity. Thereby, calcium currents are identified as key players of at least two molecular processes that mediate the change in synaptic strength by synaptic plasticity (Kandel et al., 2000; Lüscher and Malenka, 2012; Korte and Schmitz, 2016). First, inside the cell, calcium triggers different pathways resulting to changes in the number and phosphorylation of AMPA receptors yielding the potentiation (strengthening) and depression (weakening) of synapses (Choquet and Triller, 2013; Huganir and Nicoll, 2013). The second process is triggered by endocannabinoids and induces depression of synapses (Sjöström et al., 2001; Sjöström et al., 2003; Nevian and Sakmann, 2006). The endocannabinoids are transmitted retrogradely from the postsynaptic neuron to the presynaptic neuron changing the neurotransmitter release capability. For this, calcium plays a crucial role (Hashimotodani et al., 2005; Maejima, 2005). These molecular findings are integrated into a theoretical model, in which the postsynaptic calcium concentration within the dendritic spine directly accounts for synaptic plasticity (Graupner and Brunel, 2012). The calcium concentration, in turn, is modulated by spike-dependent pre- and postsynaptic processes. This calcium-dependent model matches several experimental findings and represents a biologically detailed theoretical model of synaptic plasticity.

After introducing our mathematical method to derive compact models from detailed models (see section 2), we simplify the calcium-dependent model of synaptic plasticity to derive a compact model with synaptic changes dependent on pre- and postsynaptic firing rates. We repeat this procedure for different versions of the considered synaptic plasticity model as well as neuron model. For all cases, the resulting compact model reliably matches the dynamics of the detailed model indicating that, despite the mathematical simplification, the compact model captures the essentials of synaptic dynamics. Further analyses of the resulting compact models reveal, amongst others, that the dynamics of synaptic plasticity are dominated by the square of the presynaptic firing rate, which is in contrast to phenomenologically derived compact theoretical models. Thus, the here developed method enables the derivation of simple, compact, and rate-dependent models from detailed ones allowing further investigations on the network level without loss of essential details of the synaptic dynamics.

## 2. Materials and Methods

In this study, we derive a simplified, compact, firing-rate-dependent mathematical formulation of the detailed dynamics of spike-timing dependent plasticity. For this, we analyze the firing rate dependency of the detailed model and, based on this, estimate the functional relations between activity and synaptic weight changes by regression analysis. As detailed model, we consider a well-established calcium-based synaptic plasticity model, which describes a multitude of plasticity effects based on the underlying molecular dynamics of calcium currents in the postsynaptic dendritic spine (Graupner and Brunel, 2012; Graupner et al., 2016). To take the influence of network effects and postsynaptic activity dynamics into account, we focus in our analysis on three fundamental network motifs. These motifs consist of one or two different presynaptic neuronal populations connected to one postsynaptic neuron. In the following, first, we introduce the different considered motifs and the detailed models of synaptic plasticity as well as used neuron models (please see Table 1 for used parameter values). Then, we describe our generic approach to investigate the firing rate dependencies of synaptic plasticity to derive a simplified, compact mathematical description.

**Table 1:** Used values for model parameters.

**Synaptic plasticity**				**MAT-neuron**			**AEIF-neuron**		
**Parameter**	**Unit**	**Linear calcium**	**Nonlinear calcium**	**Parameter**	**Unit**	**Value**	**Parameter**	**Unit**	**Value**
τ_Ca_	ms	22.27212	18.93044	τ_m_	ms	5	τ_m_	ms	9.367
C_pre_		0.84410	0.86467	*R*	MΩ	50	*E*_*L*_	mV	-70.6
C_post_		1.62138	2.30815	*S*_0_	mV	20	Δ_*T*_	mV	2
θ_d_		1	1	α_1_	mV	30	*V*_*T*_	mV	-50.4
θ_p_		2.009289	4.99780	α_2_	mV	2	*R*	MΩ	33.33
γ_d_		137.7586	111.82515	τ_1_	ms	10	τ_*z*_	ms	144
γ_p_		597.08922	894.23695	τ_2_	ms	200	*a*	nS	4
τ	s	520.76129	707.02258	refr. time	ms	2	*b*	nA	0.0805
				α(*N*)	mV/s	400 (P2) / 200 (P3)	α(*N*)	mV/s	170 (P2) / 80.5 (P3)

*The values for the different calcium-based synaptic plasticity models are taken from Graupner et al. (2016), for the MAT-model from Kobayashi et al. (2009), and for the AEIF-model from Brette and Gerstner (2005). Please note that those values are derived from experimental data. The scaling constant α(N) was empirically determined by simulation, such that the maximal postsynaptic firing frequency stays within plausible ranges of activity (maxv(u, w) ≈ 130 Hz)*.

### 2.1. Neuronal Setups

**Figure 1:**
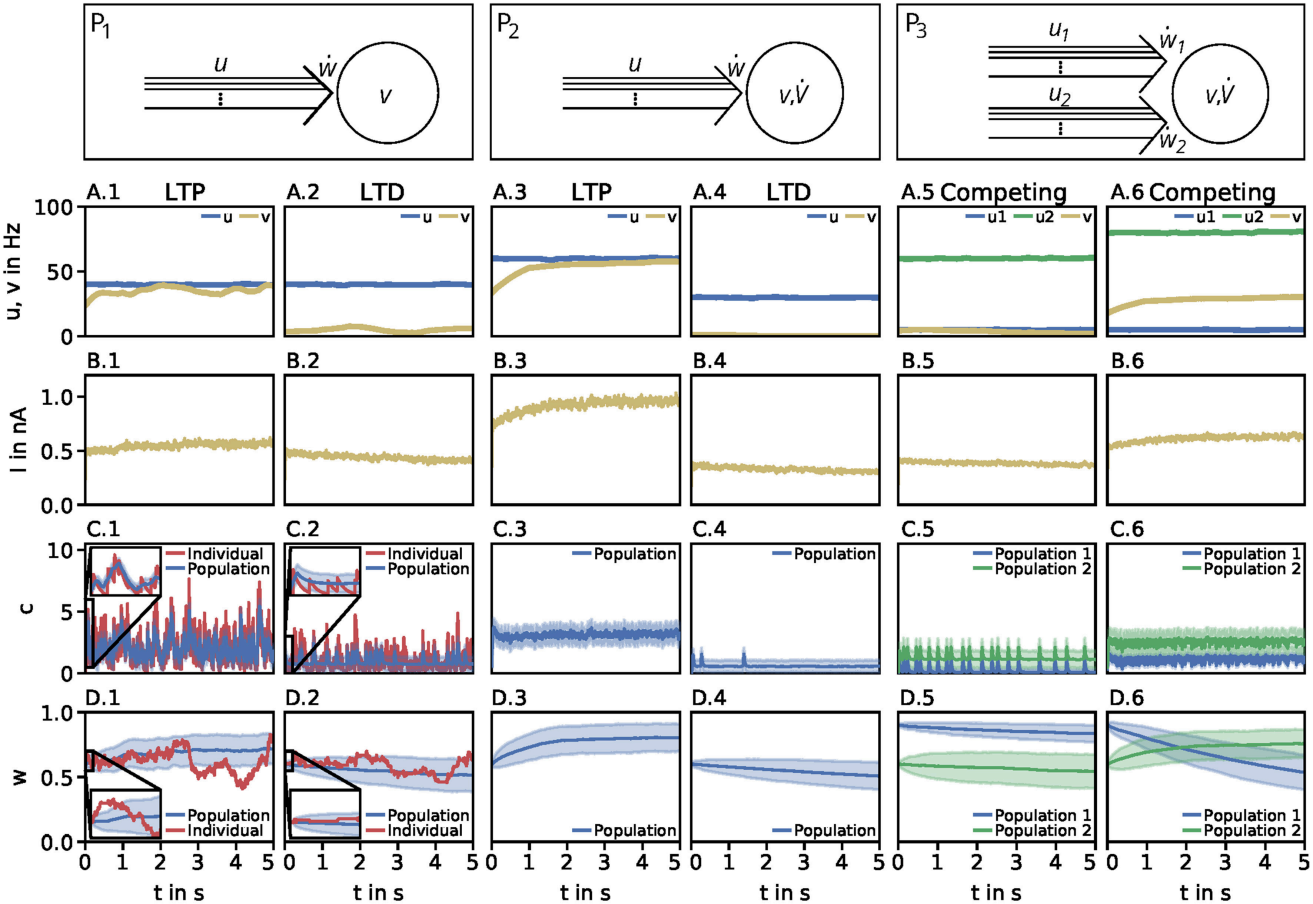
Dynamics of the detailed model of calcium-based synaptic plasticity for three different neuronal setups P_1_, P_2_, and P_3_. The different setups are shown schematically in the first row. The following rows show (second row; **A**) the average firing rates, (third row; **B**) the postsynaptic input current, (fourth row; **C**) the calcium concentration of an individual synapse using the LC-model (red) and population average (blue) with standard deviation (blue shading), and (fifth row; **D**) the synaptic weight of an individual synapse (red) and population average (blue) with standard deviation (blue shading). Columns 1 and 2: Dynamics of the P_1_-setup for initial synaptic strengths of *w*(*t*_0_) = 0.6 and pre- and postsynaptic spike-firing of *u* = *v* = 40Hz (column 1; LTP) and *u* = 40Hz and *v* = 5Hz (column 2; LTD). Columns 3 and 4: Same as in columns 1 and 2 for the P_2_-setup with presynaptic spike-firing of *u* = 60Hz (column 3; LTP) and *u* = 30Hz (column 4; LTD). Columns 5 and 6: Same as in previous columns for P_3_-setup of two, competing presynaptic populations 1 (blue) and 2 (green) with initial synaptic weights of *w*_1_(*t*_0_) = 0.9, *w*_2_(*t*_0_) = 0.6 and presynaptic spike-firing of *u*_1_ = 5Hz, *u*_2_ = 60Hz (column 5; no competition) and *u*_1_ = 5Hz, *u*_2_ = 80Hz (column 6; strong competition). In all cases the populations consist of *N* = 1, 000 synapses. The continuous presynaptic activities were obtained by averaging all *N* spiketrains and temporally filtering with an alpha function window of 100ms width. The postsynaptic activities were obtained with an alpha function window of 500ms width and using the MAT-model. The calcium and current traces were filtered with a moving average of 25ms width.

Throughout this study we consider three basic neuronal motifs or setups: a presynaptic population with independent postsynaptic neuron (P_1_; Figure 1, top left), a presynaptic population with dynamic postsynaptic neuron (P_2_; Figure 1, top middle), and two presynaptic populations with dynamic postsynaptic neuron (P_3_; Figure 1, top right).

Neuronal Population I (P_1_): This motif consists of one population of *N* = 1, 000 presynaptic neurons (single lines abstracted to one arrow) connected to one postsynaptic neuron (circle). All presynaptic neurons fire independently from each other by a Poisson process with frequency *u*. The postsynaptic neuron is active by a Poisson process with frequency *v*. Note, in this setup the firing of the postsynaptic neuron is independent of the activity of the presynaptic population, since all spike trains are generated independently by probabilistic Poisson processes. These processes can yield, at a specific point in time, different synaptic weights for different presynaptic neurons. Thus, we consider the average synaptic strength of the presynaptic population denoted by *w*.

Neuronal Population I with neuron model (P_2_): This setup is similar to the P_1_-setup. However, here, the activity of the postsynaptic neuron is not determined by a Poisson process (see P_1_), instead, we consider a biologically detailed neuron model. For generality, throughout this study we use two different neuron models; the so-called Multi-timescale Adaptive Threshold model (MAT; Kobayashi et al., 2009) and the Adaptive Exponential Integrate-and-Fire model (AEIF; Brette and Gerstner, 2005). Thus, the activity of the presynaptic population can influence the firing of the postsynaptic neuron. Consequently, the change in the postsynaptic firing rate ($\dot{v}$) depends on *u* and *w*, i.e., $\dot{v}\left(u,w\right)$, which in turn influences the dynamics of synaptic plasticity (ẇ).

Neuronal Population II (P_3_): This motif is an extension of the P_2_-setup. Here, two presynaptic populations 1 and 2 are connected to the same postsynaptic neuron with each population consisting of independently firing neurons with population-specific frequency *u*_1_ and *u*_2_, respectively. Thus, the firing of the postsynaptic neuron depends on the presynaptic firing rates and strength of the connecting synapses of both presynaptic populations ($\dot{v}\left(u_{1},u_{2},w_{1},w_{2}\right)$). Note, the only difference between both populations is the firing frequency, as all other parameters are set to be the same. Consequently, we only investigate the influence of the firing rate of the first population as results also hold for the second population.

### 2.2. Calcium-Based Synaptic Plasticity Model

The strength of an individual synapse connecting a presynaptic neuron *i* with the postsynaptic one, denoted by ρ_*i*_, underlies the following dynamic (Graupner and Brunel, 2012;Graupner et al., 2016):

(1) $$\begin{array}{l} \tau \dot{\rho_{i}}=\underset{\text{LTP}}{\underset{︸}{\gamma_{\text{p}}\left(1-\rho_{i}\right)\text{H}\left(c_{i}\left(t\right)-\theta_{\text{p}}\right)}}-\underset{\text{LTD}}{\underset{︸}{\gamma_{\text{d}}\rho_{i}\text{H}\left(c_{i}\left(t\right)-\theta_{\text{d}}\right)}}+{\text{Noise}}_{i}\left(t\right)\text{.} \end{array}$$

The first term describes the processes of long-term potentiation (LTP) at a synapse with γ_p_ being the rate of synaptic increase, θ_p_ being the potentiation threshold. *c*_*i*_(*t*) is the activity-dependent postsynaptic calcium-concentration, which is described either by a linear calcium model (LC; see section 2.2.1) or a more complex nonlinear calcium model (NLC; see section 2.2.2). H(*x*) denotes the Heaviside function. Analogously, the second term describes the processes of long-term depression (LTD) at a synapse in accordance to the parameters γ_d_ and θ_d_.

The noise term accounts for coincidental activity-dependent dynamics and non-plasticity related weight dynamics and is given by

(2) $$\begin{array}{l} {\text{Noise}}_{i}\left(t\right)=\sigma \sqrt{\tau }\sqrt{\text{H}\left(c_{i}\left(t\right)-\theta_{\text{p}}\right)+\text{H}\left(c_{i}\left(t\right)-\theta_{\text{d}}\right)}\eta_{i}\left(t\right)\text{,} \end{array}$$

where τ is the same temporal constant as in Equation 1, σ is a constant amplitude parameter, and η_*i*_(*t*) represents Gaussian white noise with mean zero and unit variance drawn for each synapse individually.

#### 2.2.1. Linear Calcium Dynamics (LC)

In the linear calcium (LC) model, the dynamics of the intracellular calcium concentration of the postsynaptic spine for each synapse, denoted as $\dot{c_{i}}$, is given by:

(3) $$\begin{array}{l} \begin{array}{l} {\dot{c}}_{i}=-\frac{c_{i}}{\tau_{\text{Ca}}}+{\text{C}}_{\text{pre}}\Sigma_{k}\delta (t-t_{k}^{i})+{\text{C}}_{\text{post}}\Sigma_{l}\delta (t-t_{l})\text{,} \end{array} \end{array}$$

where τ_Ca_ is the time constant of the calcium decay. The calcium concentration increases with pre- and postsynaptic spike times $t_{k}^{i}$ and *t*_*l*_ of amplitudes C_pre_ and C_post_, respectively. Delays between spiking events and the flow of ions are neglected as such delays only shift the independent, probabilistic spike events in the time domain. Please note, instead of Equation 3, we also consider a more complex set of equations to model the calcium dynamics as described in the following.

#### 2.2.2. Nonlinear Calcium Dynamics (NLC)

In the nonlinear calcium (NLC) model, the calcium concentration *c*_*i*_(*t*) = *c*_*i*, pre_(*t*) + *c*_*i*, post_(*t*) is determined by presynaptically evoked transients of calcium, described by

(4) $$\begin{array}{l} \begin{array}{l} {\dot{c}}_{i,\text{pre}}=-\frac{c_{i,\text{pre}}}{\tau_{\text{Ca}}}+{\text{C}}_{\text{pre}}\Sigma_{k}\delta (t-t_{k}^{i}), \end{array} \end{array}$$

and postsynaptically evoked transients of calcium given by

(5) $$\begin{array}{l} \begin{array}{l} {\dot{c}}_{i,\text{post}}=-\frac{c_{i,\text{post}}}{{\text{τ}}_{\text{Ca}}}+{\text{C}}_{\text{post}}\Sigma_{l}\delta (t-t_{l})+\xi \;\Sigma_{l}\delta (t-t_{l})c_{i,\text{pre}}\text{,} \end{array} \end{array}$$

where the quantities τ_Ca_, $t_{k}^{i}$, *t*_*l*_, C_pre_ and C_post_ are analog to the linear calcium model. The nonlinearity factor

(6) $$\begin{array}{l} \xi =\frac{2({\text{C}}_{\text{post}}+{\text{C}}_{\text{pre}})-{\text{C}}_{\text{post}}}{{\text{C}}_{\text{pre}}} \end{array}$$

couples the pre- and postsynaptic calcium concentrations (Graupner et al., 2016).

### 2.3. Neuron Model

#### 2.3.1. Multi-Timescale Adaptive Threshold Neuron (MAT)

The multi-timescale adaptive threshold (MAT) model (Kobayashi et al., 2009; Yamauchi et al., 2011) describes the postsynaptic membrane potential as a leaky integrator:

(7) $$\begin{array}{l} \tau_{\text{m}}\dot{V}=-V\left(t\right)+\text{R}I\left(t\right)\text{,} \end{array}$$

where τ_m_ is the membrane time constant, R the membrane resistance, and *I* the excitatory postsynaptic current. The excitatory postsynaptic current provides the linkage to the calcium-based plasticity model by summing the incoming spikes dependent on the synaptic weights:

(8) $$\begin{array}{l} I\left(t\right)=\alpha \left(\text{N}\right)\frac{\tau_{\text{m}}}{\text{R}}\overset{\text{N}}{\underset{i=1}{\sum }}\rho_{i}\delta \left(t-t_{k}^{i}\right)\text{.} \end{array}$$

*N* denotes the size of the presynaptic population, ρ_*i*_ the synaptic strength connecting the presynaptic neuron *i* to the postsynaptic neuron, $t_{k}^{i}$ the presynaptic spiking times, and α(*N*) is a population-size-dependent scaling constant for the current.

After each postsynaptic spike time *t*_*j*_, the spiking threshold *S* gets adapted by

(9) $$\begin{array}{l} \text{S}\left(t\right)=\underset{j}{\sum }\Delta \left(t-t_{j}\right)+{\text{S}}_{0}\text{,} \end{array}$$

with S_0_ being the resting threshold. The update of the threshold and its decay is described by multiple timescales, τ_1_ and τ_2_, and amplitudes, α_1_ and α_2_, and is given by

$$\begin{array}{l} \Delta \left(t\right)=\alpha_{1}\exp\left(-t/\tau_{1}\right)+\alpha_{2}\exp\left(-t/\tau_{2}\right)\text{.} \end{array}$$

#### 2.3.2. Adaptive Exponential Integrate-and-Fire Neuron (AEIF)

The adaptive exponential integrate-and-fire (AEIF) model (Brette and Gerstner, 2005) describes the postsynaptic membrane potential as

(10) $$\begin{array}{l} \tau \text{m}\dot{V}=-V+E_{L}+\Delta_{T}\exp\left(\frac{V-V_{T}}{\Delta_{T}}\right)-Rz+RI, \end{array}$$

where $\tau_{\text{m}}=\frac{C}{g_{L}}=CR$ is the membrane time constant composed of the membrane capacitance *C* and the leak conductance *g*_*L*_, *E*_*L*_ is the leak reversal potential, *V*_*T*_ the spiking threshold, Δ_*T*_ a slope factor, *I* the excitatory postsynaptic current as given in Equation 8, and *z* is the adaptation current determined by

(11) $$\begin{array}{l} \tau_{z}\dot{z}=a\left(V-E_{L}\right)-z\text{.} \end{array}$$

Here, τ_*z*_ denotes the adaptation time constant and *a* the subthreshold adaptation. Once a spike is triggered, the variable z is increased by an amount *b* which denotes the spike-triggered adaptation and *V* is reset to the reset potential *V*_*r*_ = *E*_*L*_.

### 2.4. Acquisition of Data, Transformation, and Analysis

**Figure 2:**
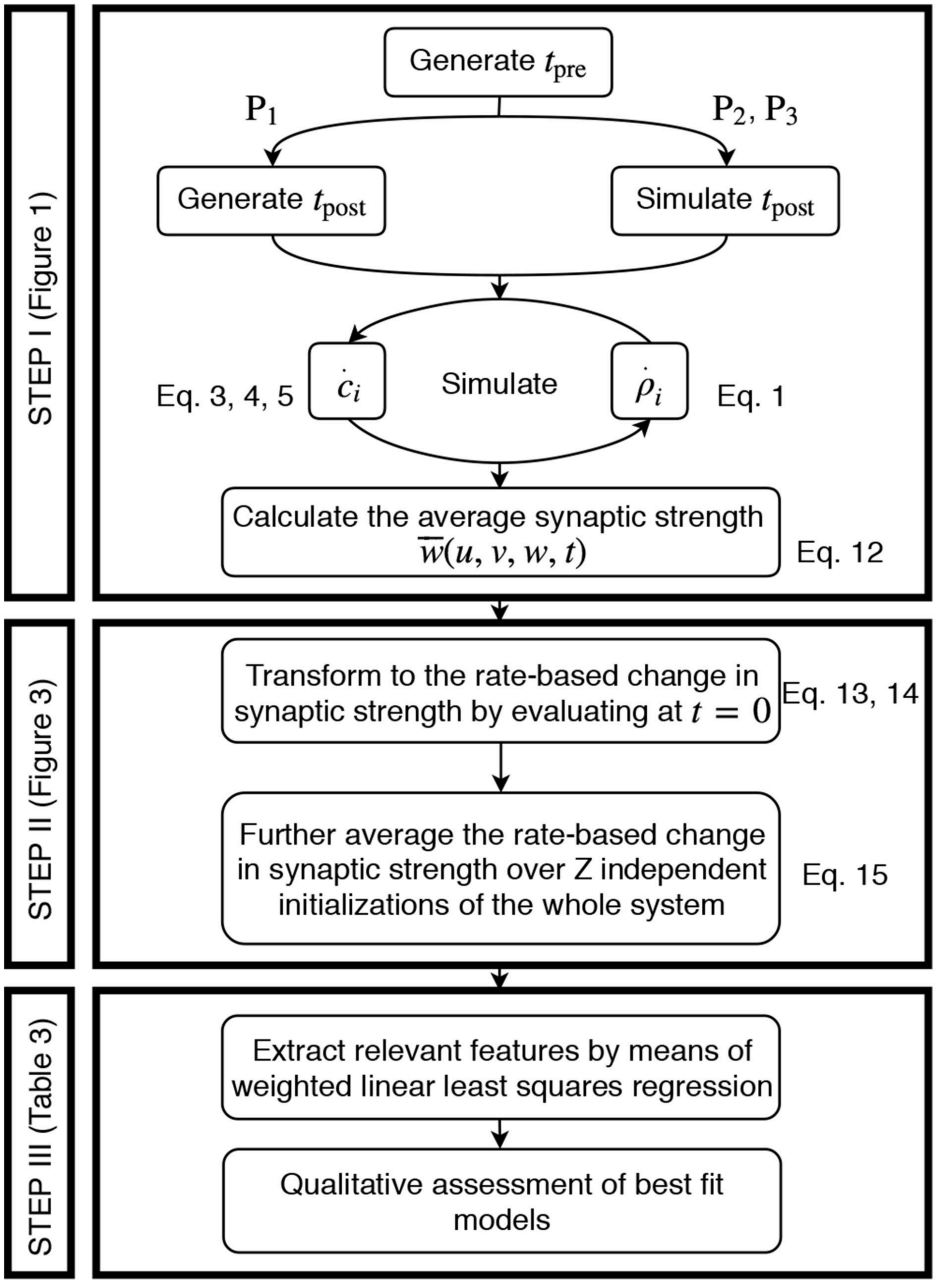
Generic approach to derive the firing rate dependencies of calcium-based STDP. Step I involves the simulation of the detailed calcium-based STDP model. Step II transforms the data obtained in Step I to rate-dependent data of synaptic plasticity. In Step III the rate-dependent data is analyzed using a regression algorithm and the results are evaluated.

The acquisition of the data resulting from the detailed model and its transformation to an activity-dependent, compact model of synaptic plasticity is carried out in a similar manner for all three different neuronal setups, two plasticity, and two neuron models. In the following, the corresponding steps taken are explained (Figure 2).

#### Step I: Simulation of Calcium-Based STDP.

We solve the differential equations of synaptic (and neuronal) dynamics numerically by the Euler method with time step Δ*t* = 0.5 ms for different presynaptic input frequencies. The parameter space of the system is given by the presynaptic firing frequency *u* (and postsynaptic activity *v* for P_1_) between 0 and 100 Hz (with a step size of 1 Hz) and the initialization of the synaptic strength *w*(*t*_0_) between 0 and 1 (in arbitrary units) with a step size of 0.05. Please see Table 2 for an overview of the analyzed parameter spaces. An exemplary time evolution of the involved quantities is shown in Figure 1 for the LC-model (section 2.2.1) with the MAT neuron model (section 2.3.1). Due to the fast time constant for the calcium concentration (τ_Ca_), the calcium concentration is initialized at zero.

**Table 2:** Mathematical notations for the different neuronal setups.

	**Postsynaptic firing**	**Change in synaptic strength**	**Parameter space**
P_1_	*v* is preset	ẇ^num^(*u, v, w*)	Ω_1_ = {(*u, v, w*):*u, v* ∈ [0, 100], *w* ∈ [0, 1]}
P_2_	*v* = *v*(*u, w*)	ẇ^num^(*u, v*(*u, w*), *w*)	Ω_2_ = {(*u, v, w*):*u* ∈ [0, 100], *v* = *v*(*u, w*), *w* ∈ [0, 1]}
P_3_	$v=v(\underline{u},\underline{w})$	${\dot{w}}_{1}^{\text{num}}(u_{1},v(\underline{u},\underline{w}),w_{1})$	$\Omega_{3}=\{(\underline{u},v,\underline{w}):u_{1,2}\in [0,100],v=v(\underline{u},\underline{w}),w_{1,2}\in [0,1]\}$

As the activities drive the change of synaptic weights, given each pair of pre- and postsynaptic spike train, we calculate the average synaptic strength ($\bar{w}$) of all synapses (ρ_*i*_) across a period of two seconds:

(12) $$\begin{array}{l} \bar{w}\left(u,v,w,t\right)=\frac{1}{N}\overset{N}{\underset{i=1}{\sum }}\rho_{i}\left(t\right)\text{,} \end{array}$$

where *N* is the size of the presynaptic population and ρ_*i*_ is given by solving Equation 1 with corresponding calcium dynamics numerically. Alongside, the numerical simulation yields the activity of the postsynaptic neuron. The latter is preset for P_1_ or calculated as spike count over 500 ms for P_2_ and P_3_, denoted as *v*(*u, w*) for P_2_ and $v(\underline{u},\underline{w})$ for the P_3_-setup (here $\underline{x}$ indicates a vector). However, the resulting $\bar{w}\left(u,v,w,t\right)$ is analyzed further in Step II described next.

#### Step II: Transformation to Rate-Based Data for Synaptic Plasticity.

The mean synaptic strength $\bar{w}\left(t\right)$ from the numerical solution of the detailed synaptic plasticity model is further approximated by cubic splines to $\tilde{w}\left(t\right)$. By this, the result is regularized from random fluctuations and enables us to calculate a reliable numerical derivative at the initial point in time. This approximation is described by

(13) $$\begin{array}{l} \tilde{w}\left(u,v,w,t\right)={\text{Spline}}_{\text{s}=0.1}^{\text{k}=1}\left(\bar{w}\left(u,v,w,t\right)\right)\text{,} \end{array}$$

where Spline is the UnivariateSpline function in the python library scipy.interpolate with parameters *k* and *s*. Here, *k* is the degree of the smoothing spline and *s* specifies the number of knots. The routine increases the number of knots until the smoothing condition $\int_{0}^{T}{\left(\bar{w}\left(t\right)-\tilde{w}\left(t\right)\right)}^{2}\text{d}t\leq s$ is satisfied.

By considering the synaptic dynamics $\tilde{w}\left(u,v,w,t\right)$ only around the first time point *t* = 0, on the one hand, the direct time-dependency is eliminated and, on the other hand, the weight-dependency of synaptic plasticity becomes controllable by neglecting its long-term development. Instead, we scan the weight-dependency of the weight change by considering different initial values for *w*. Thus, the *change in synaptic strength of the population* can be expressed as:

(14) $$\begin{array}{l} \dot{ŵ}\left(u,v,w\right)=\dot{\tilde{w}}\left(u,v,w,t=0\right)\text{.} \end{array}$$

Please note that the resulting $\dot{ŵ}\left(u,v,w\right)$ does not significantly depend on the initial distribution of ρ_*i*_ (see Figure S1). We average the change in synaptic strength of the population over several statistically independent initializations z (total number of intializations *Z* = 100 for P_1_, P_2_ and *Z* = 38 for P_3_) of the same parameter set. By this, we obtain the final overall *change in synaptic strength* (from numerics)

(15) $$\begin{array}{l} {\dot{w}}^{\text{num}}\left(u,v,w\right)=\frac{1}{Z}\overset{Z}{\underset{z=1}{\sum }}{\dot{ŵ}}_{z}\left(u,v,w\right)\text{.} \end{array}$$

In case of P_3_, in which we consider two independent presynaptic populations, thus, *v*(*u*_1_, *u*_2_, *w*_1_, *w*_2_), we add indices in order to differentiate between both populations such that

(16) $$\begin{array}{l} {\dot{w}}_{1}^{\text{num}}\left(u_{1},v,w_{1}\right)\text{ and }{\dot{w}}_{2}^{\text{num}}\left(u_{2},v,w_{2}\right) \end{array}$$

describe the change of the average synaptic strength of population 1 and 2, respectively. As mentioned before, in the P_3_-setup both populations dynamics are similar. Therefore, we will only consider the weight changes and variables related to the first population, if not stated otherwise.

In summary, based on these definitions for all neuronal setups (Table 2), the (average) synaptic weight changes of the detailed model ẇ commonly depends on the average population firing rate *u*, the postsynaptic firing rate *v*, and the average synaptic strength *w*, which are all local variables of synaptic plasticity (Gerstner and Kistler, 2002).

#### Step III: Regression Analysis.

Given the transformation of the numerical data from spike-dependencies to firing rate dependencies (Step II), in the following, we fit our rate-based data of synaptic changes ẇ^num^(*u, v, w*) by regression analysis to obtain compact mathematical formulations of the underlying weight dynamics. Thus, we will obtain a function of the form of

(17) $$\begin{array}{l} {\dot{w}}_{ij}=f\left(u_{j},v_{i},w_{ij}\right)\text{,} \end{array}$$

with *i* being the postsynaptic and *j* the presynaptic neuron.

To obtain such a function, we fit the rate-dependent data (Step II) by a Taylor series expansion containing combinations of the involved state variables *u, v, w* (for better readability we omit the neuronal indices) with coefficients *c*_α*βγ*_. The indices α, β, and γ indicate the order of the state variables *u*, *v*, and *w* in the corresponding feature *u*^α^*v*^β^*w*^γ^:

(18) $$\begin{array}{l} \begin{array}{c} \dot{w}=c_{000}+c_{100}u+c_{010}v+c_{001}w+c_{200}u^{2}+c_{110}uv\\ +c_{101}uw+c_{020}v^{2}+c_{011}vw+c_{002}w^{2}+\cdot \cdot \cdot . \end{array} \end{array}$$

Here, we limit the amount of considered features (individual terms) to the 27 listed in **Table 4** to reduce the number of possible combinations for the regression analysis. These are all features with α, β, γ ∈ {0, 1, 2}; thus, individual state variables of these features are maximally of the second order. The choice of the second order is related to general rate-based equations used in literature, such as the Hebb rule (Hebb, 1949), the Oja rule (Oja, 1982), or the BCM rule (Bienenstock et al., 1982). In all these equations, no quantity is higher than the second order (Tetzlaff et al., 2011). Of course, higher orders or rather more features can be considered if required.

The regression algorithm, we use, is a weighted linear least squares regression and thus minimizes the weighted sum of squared residuals. The weights are inferred from the variance across the results from the *Z* independent initializations. As a measure of accuracy, the weighted coefficient of determination (*R*^2^) was determined from 5-fold cross-validation. Note that it was further unbiased with respect to the number of considered features (Theil, 1958; Willett and Singer, 1988).

#### Qualitative Analysis of Resulting Compact Models

From the procedure described above we obtain a compact model consisting of a simplified differential equation, which resembles the dynamics of the corresponding complex model. However, to provide a better understanding of the underlying properties of the resulting compact models, we will assess whether these fulfill several qualitative characteristics of synaptic plasticity identified from numerical results (here we focus on the LC-model). For this, we define nine different qualitative characteristics, which are provided in the following. Please note that these characteristics are defined such that a compact model can either fulfill them or not.

1. LTP Area: If the average synaptic weight is below a certain value, essentially for all combinations of pre- and postsynaptic frequencies, LTP dominates the synaptic changes. The LTP Area weight value is about 0.3 (ẇ(*w*) > 0 for *w* > 0.3).
2. LTD Increase: If the synaptic weight is between the LTP Area weight value and the equilibrium value of the synaptic dynamics (w^*^), the influence of LTD on the synaptic dynamics increases with larger weight values which, in turn, decreases the overall influence of LTP with LTP still being the dominant process (ẇ > 0 with ẇ(*w*_1_) > ẇ(*w*_2_) for *w*_1_ < *w*_2_).
3. LTD Area: If the synaptic weight is above the equilibrium value w^*^, LTD dominates the synaptic weight dynamics for all combinations of pre- and postsynaptic frequencies (ẇ(*w*) < 0 for *w* > w^*^).
4. Invariability: If the pre- and postsynaptic activities are below 10 Hz, synaptic changes become negligible (ẇ ≈ 0 for *u, v* < 10).
5. Curvilinearity: For increasing pre- and/or postsynaptic activities (above 10 Hz), ẇ(*u, v*) switches from a convex function to a concave function.
6. Saturation: For high levels of neuronal activity, synaptic changes (ẇ) become independent of the actual frequency of neuronal activities.
7. *w*-Behavior: For the P_1_-setup, the change of synaptic weights depends linearly on the actual synaptic weight (ẇ(*w*) ≈ *w*). For the P_2_- and P_3_-setup, ẇ(*w*) has one maximum.
8. Competition: (Only for the P_3_-setup) If one population is highly active, the connections from the second to the postsynaptic neuron experience depression via competition.
9. Steadiness: (Only for the P_3_-setup) If the activity of one population approaches a maximum level, the influence of the other population on the system dynamics is negligible.

## 3. Results

In this study, we develop a simple, compact mathematical model by inferring the neuronal firing rate dependency of synaptic plasticity from a detailed, calcium-based synaptic plasticity model (Graupner and Brunel, 2012; Graupner et al., 2016). For this, we consider three different neuronal setups of one (P_1_, P_2_) or two (P_3_) presynaptic neuronal population(s) connected onto one postsynaptic neuron and analyze the dependencies of synaptic changes (ẇ) on the (average) pre- and postsynaptic neuronal activities (*u, v*) and the corresponding synaptic weight (*w*). For generality, we consider two different models of synaptic plasticity and two different neuron models (see section 2). In other words, for a given neuron model, plasticity model, and neuronal setup, first, we simulate the dynamics of the detailed plasticity model given different levels of pre- and postsynaptic activities. Then, we transfer the data from spiking-based to rate-based and, finally, fit the activity-dependencies by regression analysis. This analysis provides the synaptic changes given different terms or features of pre-, postsynaptic activity, and the synaptic weight and is repeated for other combinations of models and setups. For details please see section 2. Please note that, in the following, we will focus on synaptic plasticity with linear calcium dynamics (LC-model; section 2.2.1) with the MAT neuron model (section 2.3.1) to explain the basics of our approach and results.

**Figure 3:**
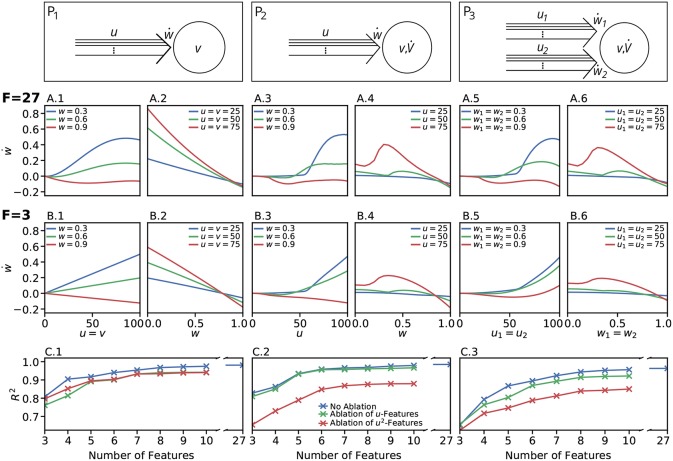
Firing rate-dependent synaptic plasticity dynamics for the three different neuronal setups P_1_ (left) P_2_ (middle), and P_3_ (right). Data for the LC-model with the MAT neuron model are shown. **(A)** Activity-based change in synaptic strength for the reference fit with 27 features (*F* = 27) for predefined initial synaptic weights (odd panels) and neuronal activities (even panels) for each neuronal setup, respectively. Deviations from simulated data are negligible. **(B)** Same as in **(A)** for the best model with three features (*F* = 3). **(C)** For a wide variety of number of features the corresponding models (indicated by the coefficient of determination *R*^2^) match the simulated data describing the dependencies of synaptic weight changes on activities and current weight.

We restricted the regression analysis by considering maximal second order terms resulting in 27 different features of *u, v, w* each individually weighted (see **Table 4** for all considered features). Thus, for all neuronal setups, we searched for the most relevant combinations of 27 features that fit best the simulation-based dependencies evaluated by calculating the coefficient of determination between fit and simulation data from 5-fold cross-validation in the four-dimensional space of ẇ, *w, u, v* (see Figure 3A for different intersections). The resulting best fits serve as *reference estimator* of the corresponding setup. The reference estimators reached accuracies of *R*^2^ = 0.981 for the P_1_-setup (Figures 3 A.1,A.2,C.1), *R*^2^ = 0.986 for the P_2_-setup (Figures 3 A.3,A.4,C.2), and *R*^2^ = 0.963 for the P_3_-setup (Figures 3A.5,A.6,C.3). To compare the resulting estimators more qualitatively with the simulation data, we defined nine characteristics of the simulated synaptic weight changes and analyzed whether the resulting estimators or compact models match these (see Material and Methods and Table 3). The reference estimators meet all characteristics (except Saturation and, additionally, Steadiness in P_3_) and, thus, the reference estimators basically describe the dynamics of the detailed, calcium-based synaptic dynamics. However, common simple models of synaptic plasticity consist of a small number of individual terms (Gerstner and Kistler, 2002; Tetzlaff et al., 2011; Gerstner et al., 2014). Thus, we repeated the regression analysis searching for the combination of *three* features fitting best the data (Figure 3B) and compared the most accurate *three-feature-estimators* with the corresponding *reference estimator*. As expected, the lower number of considered features fits the data less accurate (see Figure 3C), however, the resulting differences remain quantitative (large values of *R*^2^) and qualitative small (compare Figure 3B with A).

**Table 3:** Assessment of best fit models with nine qualitative characteristics.

**Setups**	**P_**1**_**	**P_**2**_**	**P_**3**_**	**P_**1**_**	**P_**2**_**	**P_**3**_**
***R^**2**^***	**0.810**	**0.822**	**0.652**	**0.981**	**0.986**	**0.963**
***${\text{R}}_{U}^{2}$***	**-**	**-**	**-**	**0.967**		
**Estimator**	**(Equation 19)**	**(Equation 20)**	**(Equation 21)**	**Reference (*****F*** **= 27)**		
Quality						
1 LTP Area	✓	✓	✓	✓	✓	✓
2 LTD Increase	✓	✓	✓	✓	✓	✓
3 LTD Area	✓	✓	✓	✓	✓	✓
4 Invariability	✗	✓	✓	✓	✓	✓
5 Saturation	✗	✗	✗	✗	✗	✗
6 Curvilinearity	✗	✗	✗	✓	✓	✓
7 *w*-Behavior	✓	✓	✓	✓	✓	✓
8 Competition	-	-	✗	-	-	✓
9 Steadiness	-	-	✗	-	-	✗

***(Left)** Assessment of the best individual model for each neuronal setup and **(Right)** the reference fit*.

In more detail, starting with the P_1_-setup consisting of one presynaptic population connected to a postsynaptic neuron of clamped activity, the most accurate three-feature-estimator (Figures 3B.1,B.2) is given by

(19) $$\begin{array}{l} {\text{P}}_{\text{1}}\text{-setup: }\dot{w}=c_{010}v+c_{011}vw+c_{102}uw^{2} \end{array}$$

with an accuracy of *R*^2^ = 0.810 (with weighting *c*_α*βγ*_ of feature *u*^α^*v*^β^*w*^γ^; $c_{010}=0.007832\pm 5\cdot 10^{-6}$, $c_{011}=-0.009186\pm 8\cdot 10^{-6}$, $c_{102}=-0.000989\pm 4\cdot 10^{-6}$). This model fulfills all linear and nonlinear characteristics except the nonlinear characteristics of Invariability, Saturation, and Curvilinearity (Table 3).

Next, the most accurate three-feature-estimator for the P_2_-setup (Figures 3B.3,B.4), which is a presynaptic population connected to a postsynaptic neuron with dynamic postsynaptic activity, is

(20) $$\begin{array}{l} {\text{P}}_{\text{2}}\text{-setup: }\dot{w}=c_{200}u^{2}+c_{110}uv+c_{202}u^{2}w^{2} \end{array}$$

with an accuracy of *R*^2^ = 0.822 (with $c_{200}=0.0001900\pm 10^{-7}$, $c_{110}=0.0000527\pm 3\cdot 10^{-7}$, $c_{202}=-0.0001197\pm 4\cdot 10^{-7}$) and fulfilling the majority of the characteristics correctly except Saturation and Curvilinearity (Table 3). Finally, the best three-feature-estimator for the P_3_-setup (Figures 3B.5,B.6), which is similar to the P_2_-setup except that it consists of two presynaptic populations, is given by

(21) $$\begin{array}{l} \begin{array}{l} {\text{P}}_{\text{3}}\text{-setup: }\dot{w}=c_{200}u^{2}+c_{210}u^{2}v+c_{202}u^{2}w^{2} \end{array} \end{array}$$

with an accuracy of *R*^2^ = 0.652 (with $c_{200}=0.0000227\pm 2\cdot 10^{-9}$, $c_{210}=0.0000004\pm 1\cdot 10^{-10}$, $c_{202}=-0.0000789\pm 7\cdot 10^{-9}$) and depicts five of nine characteristics correctly (all except Saturation, Curvilinearity, Steadiness, and Competition; Table 3).

Although the three-feature-estimators do not match the nonlinear aspects (e.g., Curvilinearity) of the detailed synaptic plasticity dynamics, the resulting rules (Equations 19–21) reliably match the basics of synaptic plasticity, for instance the regimes of LTP and LTD. Thus, they are representing simple, compact mathematical models of synaptic plasticity specifically for each setup.

Given the different models with numbers of features ranging from three to ten (Figure 3C), we analyzed the consistency of each feature within all the eight best estimators per setup to derive which features are generally powerful in describing synaptic plasticity. For the P_1_-setup, the accuracies of these estimators range from 0.810 for three features to 0.975 for ten features (Figure 3C.1). For the setups P_2_ and P_3_, the accuracies lie in [0.822, 0.979] and [0.652, 0.956], respectively (Figures 3C.2,C.3).

Next, we repeated these analyses for all models and derived the absolute frequency for each feature occurring in the eight best estimators (Table 4). For the P_1_-setup the most frequently occurring features are *uv*, *uw* and *vw*. The Hebbian correlation terms (*uv* and *uvw*) are also often present in the P_2_- and P_3_-setup. Interestingly, besides the Hebbian terms, for all setups and models, the *u*^2^- and *u*^2^*w*-feature occur frequently. This suggests that the square of the presynaptic firing rate is an important component influencing synaptic weight dynamics. To verify this finding, we excluded all features having a *u*^2^-term and derived new estimators. These estimators match the data from the detailed model less accurately (red in Figure 3C) than the estimators with *u*^2^-terms (blue). Note that, if for instance all terms being linear in *u* are excluded (green), the *R*^2^-values of the resulting estimators are similar to the complete estimators emphasizing the importance of the *u*^2^-terms. Thus, we conclude a general trend toward a significant correlation between synaptic plasticity and the square of presynaptic activity, which is not often considered in literature.

**Table 4:** Number of occurrences of each feature for the eight best estimators for each setup and different models.

		**P**_****1****_		**P**_****2****_			P_**3**_		
		**LC**	**NLC**	**LC + MAT**	**NLC + MAT**	**LC + AEIF**	**LC + MAT**	**NLC + MAT**	**LC + AEIF**
1	1	-	-	-	-	-	-	-	-
2	*u*	2	-	4	-	5	3	-	6
3	*v*	8	-	4	4	1	-	-	-
4	*w*	-	-	-	-	-	-	-	-
5	*u*^2^	7	-	8	-	8	8	-	8
6	*uv*	2	8	4	8	7	7	3	7
7	*uw*	4	6	2	-	2	-	-	-
8	*v*^2^	-	6	-	3	2	5	-	-
9	*vw*	8	7	4	1	5	6	6	3
10	*w*^2^	-	-	-	-	-	-	-	-
11	*u*^2^*v*	-	2	2	2	-	1	5	1
12	*u*^2^*w*	5	6	7	6	6	7	2	7
13	*uv*^2^	1	1	-	3	3	3	5	2
14	*uvw*	1	6	5	6	1	-	-	3
15	*uw*^2^	2	-	-	-	-	-	-	-
16	*v*^2^*w*	-	-	3	3	3	-	-	-
17	*vw*^2^	-	-	4	-	-	1	2	4
18	*u*^2^*v*^2^	3	3	2	-	-	3	4	4
19	*u*^2^*vw*	-	-	1	3	-	-	3	-
20	*u*^2^*w*^2^	-	-	1	-	2	1	6	2
21	*uv*^2^*w*	1	2	-	3	3	2	5	-
22	*uvw*^2^	3	1	-	-	2	-	-	-
23	*v*^2^*w*^2^	3	-	1	2	1	-	3	-
24	*u*^2^*v*^2^*w*	-	1	-	3	-	1	4	1
25	*u*^2^*vw*^2^	1	1	-	-	-	-	1	2
26	*uv*^2^*w*^2^	-	1	-	1	1	1	-	-
27	*u*^2^*v*^2^*w*^2^	1	1	-	4	-	3	3	2

*For each setup-model combination, the three most occurring features are highlighted in gray. LC: linear calcium model (section 2.2.1); NLC: non-linear calcium model (section 2.2.2); MAT: multi-timescale adaptitive threshold neuron model (section 2.3.1); AEIF: adaptive exponential integrate-and-fire neuron model (section 2.3.2)*.

After deriving the best compact model for each setup (Equations 19–21), next, we will derive the model which most precisely describes synaptic plasticity regarding all three different neuronal setups. For that purpose we are focusing on the LC-model with the MAT neuron model. We consider a unified accuracy measure $R_{U}^{2}$ being the mean over all individual accuracies minus their standard deviation. Subtracting the standard deviation is meant to avoid a bias with respect to a certain setup.

In the following, we will discuss the three most accurate unified estimators resulting from this analysis (Table 5): The first most accurate unified estimator is

(22) $$\begin{array}{l} \dot{w}=c_{200}u^{2}+c_{210}u^{2}v+c_{201}u^{2}w \end{array}$$

with $c_{200}=0.0000228\pm 4\cdot 10^{-9}$, $c_{210}=0.0000004\pm 2\cdot 10^{-10}$, $c_{201}=-0.0000789\pm 2\cdot 10^{-8}$. Please note that the *c*_α*βγ*_-values for all unified estimators are derived from a 5-fold cross-validation across all setups and models.

**Table 5:** Assessment of the best three unified models describing the collective dynamics of all three neuronal setups with nine qualitative characteristics.

**Setups**	**P_**1**_**	**P_**2**_**	**P_**3**_**	**P_**1**_**	**P_**2**_**	**P_**3**_**	**P_**1**_**	**P_**2**_**	**P_**3**_**
***R^**2**^***	**0.629**	**0.677**	**0.626**	**0.604**	**0.739**	**0.652**	**0.798**	**0.601**	**0.630**
***${\text{R}}_{U}^{2}$***	**0.620**			**0.610**			**0.599**		
**Estimator**	**(Equation** **22)**			**(Equation** **23)**			**(Equation** **24)**		
Quality									
1 LTP Area	✓	✓	✓	✓	✓	✓	✓	✓	✓
2 LTD Increase	✗	✓	✗	✗	✗	✗	✓	✓	✓
3 LTD Area	✓	✗	✗	✓	✓	✗	✓	✓	✓
4 Invariability	✓	✓	✓	✓	✓	✓	✓	✓	✓
5 Saturation	✗	✗	✗	✗	✗	✗	✗	✗	✗
6 Curvilinearity	✗	✗	✗	✗	✗	✗	✗	✗	✗
7 *w*-Behavior	✓	✓	✗	✗	✓	✓	✓	✓	✓
8 Competition	-	-	✗	-	-	✗	-	-	✗
9 Steadiness	-	-	✗	-	-	✗	-	-	✗

*Only data for the LC-model with the MAT neuron model are shown*.

The second estimator is

(23) $$\begin{array}{l} \dot{w}=c_{200}u^{2}+c_{210}u^{2}v+c_{202}u^{2}w^{2} \end{array}$$

with $c_{200}=0.0000360\pm 4\cdot 10^{-9}$, $c_{210}=0.0000004\pm 2\cdot 10^{-10}$, $c_{202}=-0.0000786\pm 2\cdot 10^{-8}$. Both estimators or models contain the features *u*^2^, *u*^2^*v* and only differ slightly in their stabilizing features *u*^2^*w* and *u*^2^*w*^2^ (*c*_201_, *c*_202_ < 0). Quantitatively, they best match the neuronal setup P_2_. Qualitatively, they fail in correctly describing several characteristics of synaptic plasticity (Table 5).

**Figure 4:**
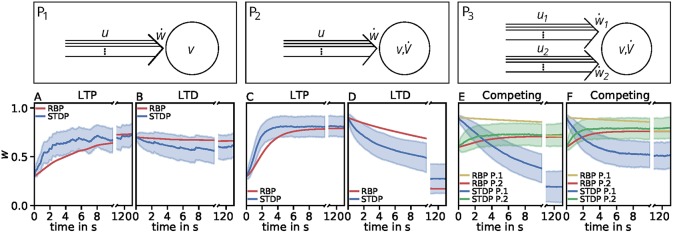
Comparison of spike-timing dependent synaptic plasticity with rate-based synaptic plasticity according to the third unified estimator (Equation 24). Here, we show the results using the LC-model with the MAT neuron model. **(A,B)**: Dynamics of P_1_ for global initial synaptic strengths of *w* = 0.3 and pre- and postsynaptic spike-firing of *u* = *v* = 35Hz (**A**; LTP) and *w* = 0.7 and *u* = *v* = 20Hz (**B**; LTD). The rate-based plasticity (RBP) is illustrated in red whereas the average spike-timing dependent plasticity (STDP) is illustrated in blue. **(C,D)** Same as in **(A,B)** for P_2_ with global initial synaptic strengths of *w* = 0.3 and presynaptic spike-firing of *u* = 65Hz (**C**; LTP) and *w* = 0.9 *u* = 36Hz (**D**; LTD). The rate-based plasticity (RBP) is illustrated in red whereas the average spike-timing dependent plasticity (STDP) is illustrated in blue. **(E,F)** Same as in **C,D** for competitive behavior in P_3_ of two presynaptic populations 1 (blue) and 2 (green) with initial synaptic weights of *w*_1_ = 0.9, *w*_2_ = 0.6 and presynaptic spike-firing of *u*_1_ = 5Hz, *u*_2_ = 75Hz (E; no competition) and *u*_1_ = 5Hz, *u*_2_ = 90Hz (**F**; strong competition). The rate-based plasticities for populations 1 and 2 are illustrated in yellow and red respectively. The corresponding average spike-timing dependent plasticity is illustrated in blue and green. In all cases the populations consist of *N* = 1, 000 synapses.

On the other hand, the third most accurate unified estimator contains Hebbian terms (*uv* and *uvw*) as well as a stabilizing one *uw*^2^ with *c*_102_ < 0:

(24) $$\begin{array}{l} \dot{w}=c_{110}uv+c_{111}uvw+c_{102}uw^{2} \end{array}$$

with $c_{110}=0.0001272\pm 3\cdot 10^{-8}$, $c_{111}=-0.0001444\pm 4\cdot 10^{-8}$, $c_{202}=-0.0011699\pm 2\cdot 10^{-7}$. Interestingly, this estimator matches best the P_1_-setup and it qualitatively generalizes better than the two most accurate unified estimators. It also correctly depicts diverse characteristics of synaptic plasticity. In addition, this estimator best captures the dynamics considering the nonlinear calcium dynamics (Table 6). We simulated the activity-dependent synaptic plasticity dynamics of this estimator (compact model) and compared it with the detailed, calcium-dependent model for the different setups using the LC-model with MAT neuron model (Figure 4). Overall, the compact model matches the dynamics of the complex model especially in the long-term dynamics for LTP as well as LTD. Thus, the long-term dynamics of the rate-based model converge to the synaptic value of spike-timing-dependent plasticity.

**Table 6:** *R*_2_-values for the unified estimators across all models.

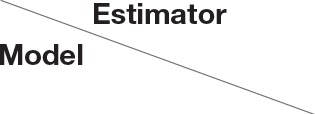	**${\text{c}}_{200}u^{2}+c_{210}u^{2}v+c_{201}u^{2}\text{w}$ (Equation 22)**	**${\text{c}}_{200}u^{2}+c_{210}u^{2}v+c_{202}u^{2}w^{2}$ (Equation 23)**	**${\text{c}}_{110}uv+c_{111}uvw+c_{102}uw^{2}$ (Equation 24)**
P1 LC	0.629	0.604	0.798
P2 LC + MAT	0.677	0.739	0.601
P3 LC + MAT	0.626	0.652	0.630
P1 NLC	0.485	0.517	0.800
P2 NLC + MAT	0.261	0.511	0.665
P3 NLC + MAT	0.317	0.427	0.644
P2 LC + AEIF	0.743	0.799	0.434
P3 LC + AEIF	0.700	0.715	0.456

**Figure 5:**
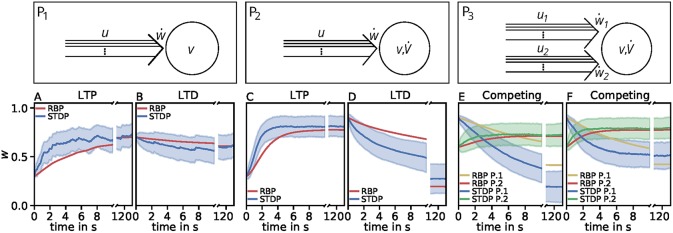
Comparison of spike-timing dependent synaptic plasticity with rate-based synaptic plasticity according to the third unified estimator (Equation 24) with the additional feature *vw*^2^. As in Figure 4, we show the results using the LC-model with the MAT neuron model. **(A,B)**: Dynamics of P_1_ for global initial synaptic strengths of *w* = 0.3 and pre- and postsynaptic spike-firing of *u* = *v* = 35Hz (**A**; LTP) and *w* = 0.7 and *u* = *v* = 20Hz (**B**; LTD). The rate-based plasticity (RBP) is illustrated in red whereas the average spike-timing dependent plasticity (STDP) is illustrated in blue. **(C,D)** Same as in **(A,B)** for P_2_ with global initial synaptic strengths of *w* = 0.3 and presynaptic spike-firing of *u* = 65Hz (**C**; LTP) and *w* = 0.9 *u* = 36Hz (**D**; LTD). The rate-based plasticity (RBP) is illustrated in red whereas the average spike-timing dependent plasticity (STDP) is illustrated in blue. **(E,F)** Same as in **(C,D)** for competitive behavior in P_3_ of two presynaptic populations 1 (blue) and 2 (green) with initial synaptic weights of *w*_1_ = 0.9, *w*_2_ = 0.6 and presynaptic spike-firing of *u*_1_ = 5Hz, *u*_2_ = 75Hz (**E**; no competition) and *u*_1_ = 5Hz, *u*_2_ = 90Hz (**F**; strong competition). The rate-based plasticities for populations 1 and 2 are illustrated in yellow and red respectively. The corresponding average spike-timing dependent plasticity is illustrated in blue and green. In all cases the populations consist of *N* = 1, 000 synapses.

However, the effect of competition between two neuronal populations is not well matched by the simple rate-based model (Figures 4E,F). If we allow a fourth feature in the estimator, the resulting compact model describes competition, too (Figure 5). This is also an example indicating that, if a specific property of the detailed model is desired, one has to consider more features to obtain a compact model which also includes this property. Similarly, if a compact model is desired which matches more accurate the detailed model, more features have to be incorporated (see e.g., Figure 3C).

In summary, our results lead to the conclusion that features of squared presynaptic activity are well describing and likely be associated to synaptic plasticity. Nevertheless, the third unified estimator (Equation 24) provides evidence that models of other features exist that better describe the qualitative linear characteristics of synaptic plasticity throughout different neuronal setups while they quantitatively perform worth.

## 4. Discussion

We developed a method to derive a simple, compact mathematical model of synaptic plasticity based on a biologically detailed, calcium-based model. In contrast to the detailed model, the resulting simple model can be used for further analytical and/or numeric analysis of, for instance, large-scale systems.

An alternative method is, for instance, to derive the cross-correlation function of neuronal firing (Brunel and Hakim, 1999; Ostojic et al., 2009) and, then, to incorporate this function into a mean-field model of synaptic plasticity (Kempter et al., 1999). This method is mathematically more accurate as our method and it can also be used to investigate the self-organization of neural circuits (Ocker et al., 2015; Tannenbaum and Burak, 2015). However, deriving the cross-correlation function of neuronal firing or the mean-field model of synaptic plasticity for diverse detailed neuron and plasticity models is very complex and, up to now, not always possible. On the other hand, the complexity of our method depends less on the considered neuron and synaptic plasticity models.

We considered two different versions of the calcium-based STDP model with parameters which where determined by Graupner et al. (2016) on slices of the visual cortex from macaque monkeys. Of course, these parameters (and also from the neuron model) could vary for different brain regions. However, with the here-developed method one can easily link these parameters to simpler models of plasticity. Given that the function of a brain region is mainly determined on the network level, by using the resulting simple model one can investigate the influence of the parameters on the functional properties of the brain regions.

Overall, we cannot guarantee that we applied the optimal weighting of the observations and thus found the best linear unbiased estimator. Technically, it could be checked if the whole covariance matrix of the N observations in Z initializations would provide a better weighting. However, this is computationally not feasible, especially for the case of P_3_ with 4.5 Mio. observations. Furthermore, we considered the estimators or models with the highest *R*^2^ while taking the next best model into account could yield other reasonable learning rules matching, for instance, more qualitative characteristics (see for instance Equation 24). If a compact model is required, which matches the detailed model with a higher accuracy, instead of three features, one can easily allow more features being present in the final estimator (Figure 3C). Furthermore, by considering features of higher order than two in Step III, the resulting final estimators could describe additional aspects of the detailed model. However, given the increased number of features, the regression procedure would require more computational resources.

The results for individual learning rules of the considered neuronal populations did not significantly match with, for instance, the BCM (Bienenstock et al., 1982) or the Oja's rule (Oja, 1982). Although the results significantly match Hebbian correlation learning, the stabilization mechanisms are different to commonly used ones. Overall, regarding the treated rate-based models, our applied methods on three different neuronal setups suggest that features from the BCM rule and Oja's rule are not the best estimators for the underlying plasticity data. From our methods, features observed to be correlated to LTP are the Hebbain term *uv* and terms, which depend on the square of the presynaptic activity such as *u*^2^*v* (opposing to *v*^2^*w* from BCM theory) and *u*^2^. Features observed to be correlated to LTD are *vw*, *u*^2^*w* (opposing to *v*^2^*w* from Oja's rule), and *uvw*. The *u*^2^-terms could imply that, on the network level, synaptic dynamics are more sensitive to variations in the inputs (*u*) than in the network activity (*v*) providing a stronger influence of the actual inputs on the overall network dynamics. However, such dynamics and the *u*^2^-dependency require further theoretical and experimental studies. For instance, by varying systematically the rate of stimulation triggering synaptic plasticity (similar to Sjöström et al., 2001), the resulting ẇ-*u*-relation could indicate a *u*^2^-dependency. Overall, the plausibility of certain terms remain unclear and corresponding molecular pathways of synaptic plasticity still have to be investigated to find pieces of evidence for these terms. However, on the one hand, our method provides a way to link complex synaptic plasticity dynamics with dynamics on the network level and, on the other hand, the individual terms from our method allow to identify important ingredients of synaptic plasticity enabling an (functional) ordering of diverse molecular pathways.

## Author Contributions

CT study design. JL data acquisition. JL, JH, and CT data analysis and manuscript written.

### Conflict of Interest Statement

The authors declare that the research was conducted in the absence of any commercial or financial relationships that could be construed as a potential conflict of interest.
