# Supplementary material for: A Theoretical Framework to Derive Simple, Firing-Rate-Dependent Mathematical Models of Synaptic Plasticity
Source: Front Comput Neurosci. 2019 May 8;13:26. doi: 10.3389/fncom.2019.00026 (PMC6517541; doi:10.3389/fncom.2019.00026)
Supplement: Supplementary file 1 [file Data_Sheet_1.PDF]

Supplementary Material

1 SUPPLEMENTARY TABLES AND FIGURES

1.1 Figures

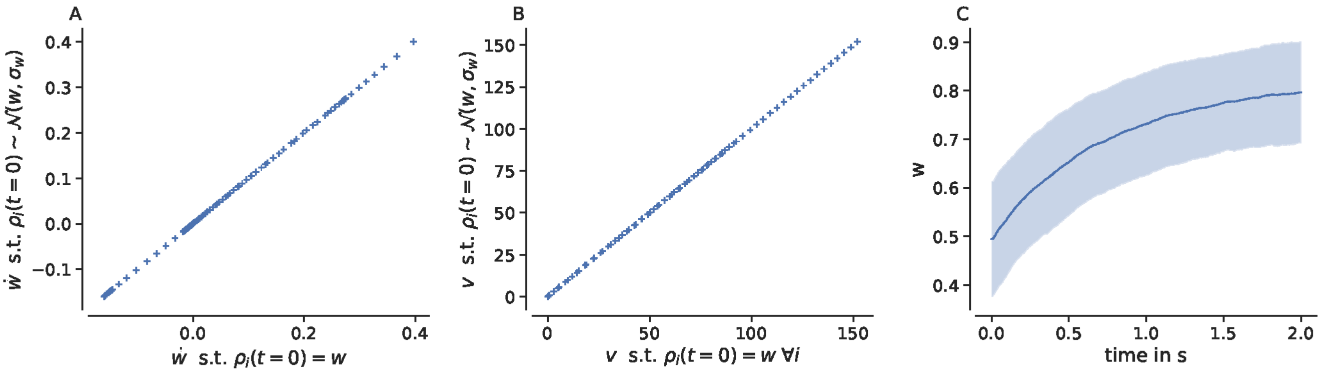

Figure S1. The effect of randomly initialized synaptic weights. Numerically derived change in average synaptic weight depend on the initialization of weights. **(A)** The average synaptic weight change $\dot{w}$ given an initial distribution of individual synaptic weights according to a normal distribution $\rho_i \sim \mathcal{N}(w, \sigma_w)$ is similar to the average synaptic weight resulting from an initialization $\rho_i = w\ , \forall i$. **(B)** Effect on the postsynaptic firing rate v, analog to (A). **(C)** Exemplary temporal evolution of the average synaptic weight with standard deviation across synapses given a normal distributed initialization. All illustrated data points underlie the P_2-setup simulated with $u \in$ `np.arange(0,101,2)` and $w \in [0, 0.5, 1]$.
